# Storm Clouds on the Horizon: On the Emerging Need to Tighten Selection Policies

**DOI:** 10.3389/fspor.2021.772181

**Published:** 2021-11-04

**Authors:** Kathryn Johnston, Lou Farah, Joe Baker

**Affiliations:** School of Kinesiology and Health Science, York University, Toronto, ON, Canada

**Keywords:** athlete selection, policy considerations, elite sport, talent selection, decision-making

## Abstract

Athlete selection is fundamental in elite sport, occurring regularly throughout an athlete's development. Research in this area reveals the accuracy of these decisions is questionable in even the most elite sport environments and athletes are increasingly disputing these decisions as unfair and punitive. As a countermeasure to these dispute and arbitration practices, many elite sport systems have created policies where coaches must outline and stand behind the criteria used for their selection decisions. Selection criteria policies have the potential to help encourage fair selection practices by holding selectors accountable to their selection criteria, but their implementation also has the potential to wrongfully nudge selectors toward developing more defendable, but less-accurate selection practices. The paper concludes with 10 suggestions to help support practitioners when implementing selection criteria.

## Storm Clouds on the Horizon for Athlete Selection

A high-performance athlete's pathway from sport entry to elite performance can be extensive, involving years and sometimes decades of effortful engagement (Baker and Young, 2014). A common occurrence throughout this pathway is evaluation of the athlete's suitability for further development and/or opportunities for more advanced levels of competition (Güllich, 2014). Ultimately, the intention behind these evaluations is to determine who has the greatest likelihood for future success. An athlete's potential for success can be assessed in many ways, from the use of intuition and “gut feelings” to complex statistical algorithms of various forms of player data. While these approaches can lead to successful athlete selection, poor accuracy rates reported in recent research suggest there is much we do not know about how selection decisions are made and how this process might be improved (Roberts et al., 2019). For example, even at professional levels – where scouting and analytics resources are abundant – the accuracy of selection is limited (Koz et al., 2012; Farah and Baker, 2020; Johnston et al., 2021). For example, a systematic review completed on the accuracy and efficacy of the draft system for the major professional sports in North America (i.e., National Basketball League, the National Football League etc.) highlighted the draft decisions are only really found to be accurate in the first and last rounds, leaving the middle rounds (often the majority of rounds) to be questionable from an economic and performance perspective (Johnston et al., 2021). No selection policy or strategy for selection decisions will ever be 100% accurate; however, without addressing voids in our understanding, selection errors and “talent wastage” will remain larger than they could be (Johnston and Baker, 2020). These errors are costly for athletes (de-selection can lead to compromised development and/or sport withdrawal; (Brown and Potrac, 2009), as well as for sport organizations (e.g., inefficient use of resources, reduced potential for international success).

It is believed much of this wastage is a result of the compounded effects of system constraints, knowledge limitations, and decision-maker errors (Den Hartigh et al., 2018b; Johnston and Baker, 2020; Till and Baker, 2020). As an example of a system constraint, many sport programs operate with strict budgets and timelines often requiring coaches to select athletes in time-pressed environments such as weekend-long training camps/try outs (Tromp et al., 2013). These timelines present challenges for coaches to gather enough information to make informed decisions about whether or not an athlete has the skills necessary (presently) and can develop within the environment the coach creates (in the future). Thus, coaches can be forced to make decisions based on snap shots of athlete performance, and in turn, may rely on gut feeling and intuition^1^ (Christensen, 2009; Jokuschies et al., 2017; Musculus and Lobinger, 2018; Roberts et al., 2021). Moreover, reduced resources could mean coaches are solely responsible for these selections without the support and guidance of other coaches and/or colleagues. Magnified by the fact that a current “recipe card” for talent does not exist, it can leave practitioners feeling confused, overwhelmed and under-supported when it comes to making accurate decisions. Last, in the case of decision-maker errors, behavioral psychologists have recognized the many different mental short cuts and blind spots present during decision-making (Kahneman and Tversky, 1973; Kahneman et al., 1991). For example, “relative age bias” is a well-documented bias within sport which describes the systematic selection of relatively older athletes presumably because of perceived performance advantages (e.g., being relatively taller or stronger than peers; Helsen et al., 2005; Wattie et al., 2015). Relative age bias, among others (for a review on potentially influential biases in sport selection see Johnston and Baker, 2020) often lead to predictable errors, highlighting our fallibilities when it comes to forming judgements under uncertainty (Kahneman et al., 1991; Lewis, 2017). For coaches, it is not a far stretch to imagine many of these same judgement and decision-making errors are present during the complex task of athlete selection (Mann and van Ginneken, 2017; Den Hartigh et al., 2018a). If these biases and errors exist, we have no idea of their prevalence or magnitude, nor how to manage their impact on selection accuracy.

Due to the potential impacts on athletes' careers, it is perhaps not surprising that athletes who have been de-selected from teams are pushing back, by appealing coaches' selection decisions. For example, an athlete with extenuating circumstances (such as sustaining a concussion) who misses a necessary event for team selection, may appeal the decision (Aburto and Bao, 2021). Multiple nations (e.g., Australia, Canada, the United Kingdom) have Dispute Resolution Systems in place to help address these concerns and to promote fairness in the selection process (Sport Dispute Resolution Centre of Canada, 2014; Dispute Resolution Services, 2021; National Sports Tribunal, 2021). In an effort to combat the rising numbers of disputes in Canada, there has been a movement toward adopting more proactive approaches to selection decisions. Specifically, efforts have shifted toward implementing more “defendable” selection processes. In this case, it would require coaches to create, and be held accountable for, their pre-determined selection criteria. For example, coaches at the National Sport Organization level in Canada are required to post their criteria for athletes and other stakeholders to review before selection camps/tryouts begin. Often, the criteria are listed in the team's policy documents and shared through the sport organization's website (see for example Athletics Canada, 2021). Coaches are then held accountable to their criteria by their governing bodies when making selections. Non-adherence could result in an athlete disputing the decision, which could subsequently lead to arbitration involving both parties' legal representation, which presents some advantages and challenges. Currently, the initiative to adopt selection criteria policies is primarily focused on National Sport Organization (NSO) teams, but it is inevitable these principles will trickle-down to the lower levels of competitive sport over time, which requires careful consideration.

## Selection Criteria Policies – the Good, the Bad, and the Risky

A shift away from using “gut feeling” and intuition, toward evidence-based approaches for selection is important for increasing accuracy in any complex decision-making environment (Kahneman and Frederick, 2002; Frederick, 2005 Hastie and Dawes, 2010). In other domains (e.g., medicine and economics), establishing and adhering to selection criteria enhances the accuracy of decision-making under uncertainty (Goldberg, 1968; Gawande, 2010; Hastie and Dawes, 2010). It is plausible the same (or similar) principles apply in the sport domain as well. The widespread adoption of these selection criteria policies could allow for a significant shift in the processes, knowledge, and awareness of selection practices from both practical and research perspectives. For instance, some coaches may regularly use selection criteria policies, while for others, it could be the first time they are ever stating their criteria explicitly and/or considered how much weight certain subjective (coaches' eye; Jokuschies et al., 2017) and objective variables hold in the decision-making process. This may, in turn, help researchers and practitioners demystify coaches' mental modeling and nudge coaches to monitor, reference, and track the efficacy of their criteria over time. As demonstrated in fields such as medicine and education, this adoption and reliance on established criteria can mitigate the influence of bias in the judgement and decision-making process, and in turn, improve accuracy (Arkes et al., 2006; Meijer et al., 2020).

Moreover, establishing clear selection criteria may also provide opportunities to examine the external factors influencing a coach's selection decision. For instance, it is possible a coach is affected by political pressures when making selection decisions, such as when parents of athletes are explicitly involved in the decision-making process (e.g., as volunteer coaches, etc.), or when they exert their influence through more implicit and covert avenues (e.g., as board members or sponsors of the team). Ultimately, establishing and adhering to clear criteria may bring attention to the potential institutional biases at play (Lewis et al., 2015).

There are risks to implementing this new standard. Hypothetically, there may be a shift toward a reliance on more “objective” characteristics (such as anthropometrics, physiological measures, and other performance indicators) that can be more easily measured and defended (in the case of an appeal). In reality, an overreliance on physical measures has already been identified as a major selection error committed by teams. Not only has this over-reliance been observed in the literature (Baker et al., 2020), there also appears to be over-reliance on fitness testing results in the selection process as shown in elite sport selection practices (e.g., in the NFL draft; Robbins, 2010; Berri and Simmons, 2011). Therefore, it is possible increasing the reliance on physical measures so they are more defendable when challenged, could accentuate this bias even further and discourage inclusion of the more subjective (i.e., coachability), less-tangible characteristics.

The use of physical measures is well- documented in the literature on talent identification to date. In fact, much of the research published focuses solely on this physical dimension of athlete performance. In a recent systematic review by Baker et al. (2020), of the nearly 2000 studies gathered for analysis, those examining athletes' physical attributes (such as physiological, anthropometrical, biomechanical, and technical sills) accounted for 40 percent of all articles. In another systematic review conducted by Johnston and Baker (2020), the authors found 60 percent of longitudinal articles in their analysis examined the physical attributes of athletes. This physical bias in the literature suggests there is much to learn about some of the under-represented areas such as training/practice histories (Ward et al., 2004; Güllich, 2019; Cowan et al., 2021) and other psychological attributes (Gould et al., 2002; Fletcher and Sarkar, 2013; Galli and Gonzalez, 2015) of elite athlete performance. This dearth is further magnified when considering most samples in talent research have been male, typically late in the athlete development pathway (late adolescence or adulthood) and focusing on mass participation sports like soccer and rugby (Baker et al., 2020; Johnston and Baker, 2020). Another important consideration is that nearly all of this research examines the accuracy and efficacy of variables assessed in isolation for athlete selection, but very little research exists on what coaches are actually using for their sources of information for selection decisions (Roberts et al., 2020; Lath et al., 2021). All this to say, there are considerable gaps in the evidence from which coaches are required to develop “evidence-based” selection criteria.

In contrast to this overreliance on objective measures, the authors suspect coaches may choose to rely more heavily on “subjective” criteria (i.e., coachability, and other psycho-social variables). According to Anshel and Lidor (2012), caution should be taken when utilizing such measures as (i) vague definitions exists for many of these psychological constructs, (ii) relatively poor predictive validity has been reported as much of the research is cross-sectional, and (iii) current limitations exist due to limited empirical research. By using more subjective variables in the criteria, coaches may create a greater degree of “wiggle room” when explaining and defending their decisions to an athlete and perhaps a dispute resolution committee. It is possible this impact could be magnified in team sports, where selections do not always equate to a collection of the “best” individual performers, but rather the best “collection” of athletes. For example, evidence suggests coaches take into consideration the “fit” of an athlete with the team (e.g., group interactions), which may have a positive effect on the training environment, but also on the competitive performance of the team (Bradbury and Forsyth, 2012). This could be mitigated, however, if subjective criteria are made explicit and are defendable, but this in itself raises challenges related to identifying reliable and valid subjective measures.

Ultimately, having a well-defined strategy and philosophy for selection will be important, provided it is viewed in the appropriate context. These types of policies, however, assume coaches can make valid and reliable early predictions about an athlete's “potential”, but the evidence supporting this assumption is not compelling (as demonstrated by the low progression rates of junior to senior levels of sport; Gulbin et al., 2013; Barreiros et al., 2014; Yustres et al., 2020; Boccia et al., 2021). A possible explanation for this inverse relationship between time and accuracy was noted by Den Hartigh et al. (2016); the non-linear and dynamic nature of sporting excellence will likely remain a challenge for improving selection accuracy and forcing coaches to implement and defend a clear set of criteria may undermine the type of research needed in this field (Abbott and Collins, 2002; Abbott et al., 2005; Den Hartigh et al., 2018a).

One way forward would be to reduce the impact of early selection decisions on athlete development. For instance, the development and implementation of stronger parallel programs (e.g., development of ‘B teams’ and/or athlete transfer initiatives), or the opportunity to participate in multiple sport programs concurrently, could provide more/alternative options for athletes to stay in the sport system, continue their development and possibly emerge further along in the athlete pathway (see Galatti et al., 2016 for an example). This, in turn, may lead to selections being made when athletes are older, when some performance traits are relatively more stable (Bragada et al., 2010; Costa et al., 2011).

## Policy and Practitioner Recommendations

Should sport systems continue to implement such selection criteria policies, caution is advised. Here, we propose 10 suggestions to help support the implementation of such policies. It is important to acknowledge, however, that many of these recommendations come with a cost, and for some organizations, the resources (human, financial and time) required to implement these changes may not be feasible. We have tried to provide a more accessible alternative where possible.

1) Consult your nation's and/or organization's dispute-resolution center. Often, these centers have a program in place to educate and support coaches and staff through the development of criteria. If such a center does not exist, it may be worthwhile to consult the organization overseeing the policy implementation.

2) Create a list of the variables you have seen in the “best” athletes you have worked with in the past. Consider athletes of a similar age and competitive level to those you are working with. Recollecting the qualities, characteristics, and traits the top performing athletes exhibit may act as a starting point for establishing your preferred criteria. However, human memory is fallible and mis-remembering or misinterpreting signals is common, so it can be helpful to consult with another member (assistant coach, staff etc.) and/or cross-reference with previously collected player data.

3) Examine your existing criteria regardless of whether your criteria are formal (written and established) or informal (in your head/never stated explicitly) and either individual (coach driven) or organizational (team, company, or institution driven). What variables have you used in the past in your own mental modeling? What evidence do you have these variables were in fact predictive? Are the variables measurable? If not, what is needed for them become more measurable?

4) Manage and analyze data effectively. Often coaches have ongoing collections of athlete data (fitness testing, demographic information, workload measures etc.) and managing those data and performing appropriate analyses can be a resource-intensive process. Where possible, it could be helpful to consult with a data scientist to help sift through previous and current data and provide an additional perspective on the types of analyses that can be used when interpreting the data. If organizations do not have the resources to access this level of expertise, exploring other options (e.g., partnering with sport science departments in post-secondary instructions) may provide some level of support under a tight budget. This approach can also assist your decision-making by integrating computer-based modeling (Owusu, 2007). Blending the best of technology and human thinking and processing may result in better decisions as the impact of the “human effect” is moderated (i.e., influence of mood, fatigue, etc.).

5) Challenge your beliefs. Recognize the influence your assumptions and biases play in predictions. By acknowledging the ways you and your team may distort and interpret signals you receive, you will help to mitigate their effects (Taleb, 2007; Silver, 2012). For instance, having an awareness of how the language being used may indicate a bias present, and could help illuminate the points of entry for that bias (e.g., identifying what information is feeding the bias). After acknowledging the presence of biased language and their points of entry, try to work to counteract the bias by searching for contradictory evidence instead of confirmatory evidence. It is often much easier to find information that corroborates our beliefs than challenges them, as captured in the bias called the ‘confirmation bias (Nickerson, 1998).

6) Consider the “measurability” and reliably of your variables. What has been researched in a rigorous and scientific way (either through the peer review system or internal research opportunities)? Seek research syntheses (meta-analyses, systematic reviews, narrative reviews) for your sport, age, and competition level to provide a less-biased approach to narrow in on which variables (and the types of measurement tools) to use. Of particular value are systematic reviews and meta-analyses which are known to provide reliable and (at least in theory) less-biased information in a given discipline. While they do not necessarily market their ability to solve practical questions, they may help researchers and practitioners by highlighting, evaluating and summarizing knowledge in what can often feel like an overwhelming volume of literature (Yuan and Hunt, 2009). In cases where empirical research is inaccessible, or lacks interpretability, consult a sport affiliated research institution to aid in gathering and disseminating research, where possible. An example of this is the Sport Information Resource Center (*The Sport Information Resource Center*, n.d., SIRC, 2021), which offers sport organizations “literature review services” tailored to their needs. If such center does not exist, consult with a local library or post-secondary library service to see what services may be offered.

7) Consult with a more neutral third party. By speaking through your decision-making approaches to another person, it can help to provide a different perspective, to challenge beliefs and strategies, and add another level of rigor to the process. In the case of a more experienced and knowledgeable “neutral third-party consultant”, this person can ask questions that you or your colleagues may not have considered. For a less-experienced and less knowledgeable consultant, this person may help by asking questions that may seem obvious, forcing you to question longstanding, habitual practices and challenge your sources of information (aka help reduce the curse of knowledge, Pinker, 2015). In a similar vein, creating mentorship and partnership opportunities within your coaching systems may be helpful for less-experienced coaches to learn from their more-experienced counterparts. Having a reliable and trustworthy mentor may act as a “sounding board” to help you evaluate practices and discuss the strengths and limitations of your proposed approaches.

8) Track your progress. By calibrating outcomes for feedback (e.g., out of all the times you said there was a 40% chance, how often did that actually occur?) you are able to track your performance as a coach (Tetlock and Gardner, 2016). Ask yourself - how am I doing over time (i.e., 1 year, 5 years etc.)? Finding ways to express and quantify uncertainty in predictions by reporting a margin of error will be helpful for increasing accuracy (Phillips, 2003; Tetlock and Gardner, 2016). The two obvious limitations are (a) this takes time (likely years) to determine which athletes have progressed in the system and which athletes have not, and (b) only your accuracy for those being selection (as opposed to those being de-selected) can be measured. Until there are parallel systems in place for athletes to continue training, tracking the performance of those who are de-selected will remain an inherent barrier.

9) Extend observation periods before selection. As noted, snap-shot judgements can lead to abrupt and impulse-driven decisions which are known to be less-accurate. Determine if more points of interaction are feasible within your current system. This can decrease the risk of overvaluing or undervaluing an athlete's ability based on an uncharacteristic superior or inferior performance. More exposure to an athlete's performance overtime will increase the chance of gathering a more accurate representation of his/her skills and abilities (Tetlock and Gardner, 2016).

10) Recognize your limitations. Acknowledge if you cannot make a good prediction, it is sometimes harmful to pretend you can (Silver, 2012).

The implementation of a selection criteria policy in Canada may reflect gathering storm clouds on the horizon, with implications for many nations' athlete development systems. These types of policies have both immediate (explaining current decision-making) and long-term implications (the consequences of poor decisions early in the pathway have repercussions later). These implications should be carefully examined as the strategy presents a unique opportunity for researchers, coaches, and policymakers to identify the limitations and weaknesses in current approaches. If they do not take advantage of the shifting landscape in their sport, they may soon find themselves having to stand behind the “status quo”.

## Data Availability Statement

The original contributions presented in the study are included in the article/supplementary materials, further inquiries can be directed to the corresponding author/s.

## Author Contributions

KJ, LF, and JB contributed to conception and design of the study. All authors contributed to manuscript revision, read, and approved the submitted version.

## Funding

Funder - Social Sciences and Humanities Research Council of Canada; Sport Canada Award # - 435-2021-0944.

## Conflict of Interest

The authors declare that the research was conducted in the absence of any commercial or financial relationships that could be construed as a potential conflict of interest.

## Publisher's Note

All claims expressed in this article are solely those of the authors and do not necessarily represent those of their affiliated organizations, or those of the publisher, the editors and the reviewers. Any product that may be evaluated in this article, or claim that may be made by its manufacturer, is not guaranteed or endorsed by the publisher.

## Footnotes

^1^There are of course, exceptions to this intuitive strategy where coaches implement decision-making rules and assign certain weights to certain athletic skills even in time-compressed situations (see Musculus and Lobinger, 2018).
